# Supplementary material for: Chance promoter activities illuminate the origins of eukaryotic intergenic transcriptions
Source: Nat Commun. 2023 Apr 1;14:1826. doi: 10.1038/s41467-023-37610-w (PMC10067814; doi:10.1038/s41467-023-37610-w)
Supplement: Supplementary file 1 — Supplementary Information [file 41467_2023_37610_MOESM1_ESM.pdf]

Supplementary Materials of “Chance promoter activities illuminate the origins of eukaryotic intergenic transcriptions” by Xu *et al.*

The supplementary materials include:
Supplementary Tables S1-S5
Supplementary Figures S1-S21

Table S1. Tested genomic locations for integration of the positive and negative controls.

No.	Tested genomic locations*	Corresponding CRISPR/Cas9 targeting sequences
1	Chr. XIII 674813-675763	ACCGTCATAGCGTTATACGT
2	Chr. IV 1502563-1503124	TGGAGTATAATACTGAAAGA
3	Chr. XI 2797-3146	TACTAATTACAAGCCTCCCA
4	Chr. XV 79704-80285	TAATCAGTCTAACACCCCGG
5	Chr. IX 426200-427000	ATTATCAGCGATTATTCAGC

*The location in Chr. IV was chosen for integration of the random promoter cassette.

Table S2. Correspondence between chance promoter activities and native gene expression levels

Expression percentile of barcodes	Expression percentile of native genes in SCD	Expression percentile of native genes in YPD
10th	0th	9.93^{th}
20th	1.77^{th}	11.85^{th}
30th	4.91^{th}	13.28^{th}
40th	6.43^{th}	14.54^{th}
50th	7.51^{th}	15.64^{th}
60th	8.39^{th}	16.39^{th}
70th	9.07^{th}	17.28^{th}
80th	10.39^{th}	18.52^{th}
90th	12.26^{th}	20.55^{th}
100th	85.72^{th}	85.09^{th}

Table S3. Fraction of intergenic regions whose expressions cannot be explained by chance promoter activities

YPD		SCD	
Considering the maximal expression among windows of an intergenic region	Considering the top 5th percentile in expression among windows of an intergenic region	Considering the maximal expression among windows of an intergenic region	Considering the top 5th percentile in expression among windows of an intergenic region
2.9%	2.7%	4.9%	4.3%

Table S4. ***P*****-values from two-tailed Wilcoxon rank-sum tests of the equality of the expression levels of neighboring genes of intergenic regions whose expressions are unattributable to chance promoter activities and those of the rest of intergenic regions.**

YPD		SCD	
Considering the maximal expression among windows of an intergenic region	Considering the top 5th percentile in expression among windows of an intergenic region	Considering the maximal expression among windows of an intergenic region	Considering the top 5th percentile in expression among windows of an intergenic region
0.91	1.00	0.60	0.90

Table S5. Results from the combined analysis of the three biological replicates and those from individual analysis of each replicate.

	YPD				SCD			
	Combined	Replicate 1	Replicate 2	Replicate 3	Combined	Replicate 1	Replicate 2	Replicate 3
% random promoters with significant promoter activities	63.20	49.09	62.11	51.04	41.35	33.72	33.05	33.51
% random promoters with significantly higher promoter activities than the reference	0.024	0.049	0.19	0.035	0.029	0.047	0.056	0.049
% intergenic windows unexplainable by chance promoter activities	1.7	1.3	0.6	1.6	7.6	4.3	3.6	4.5
% intergenic windows unexplainable by chance promoter activities and neighboring gene expressions	1.1	0.9	0.5	1.1	5.4	2.6	2.3	3.1

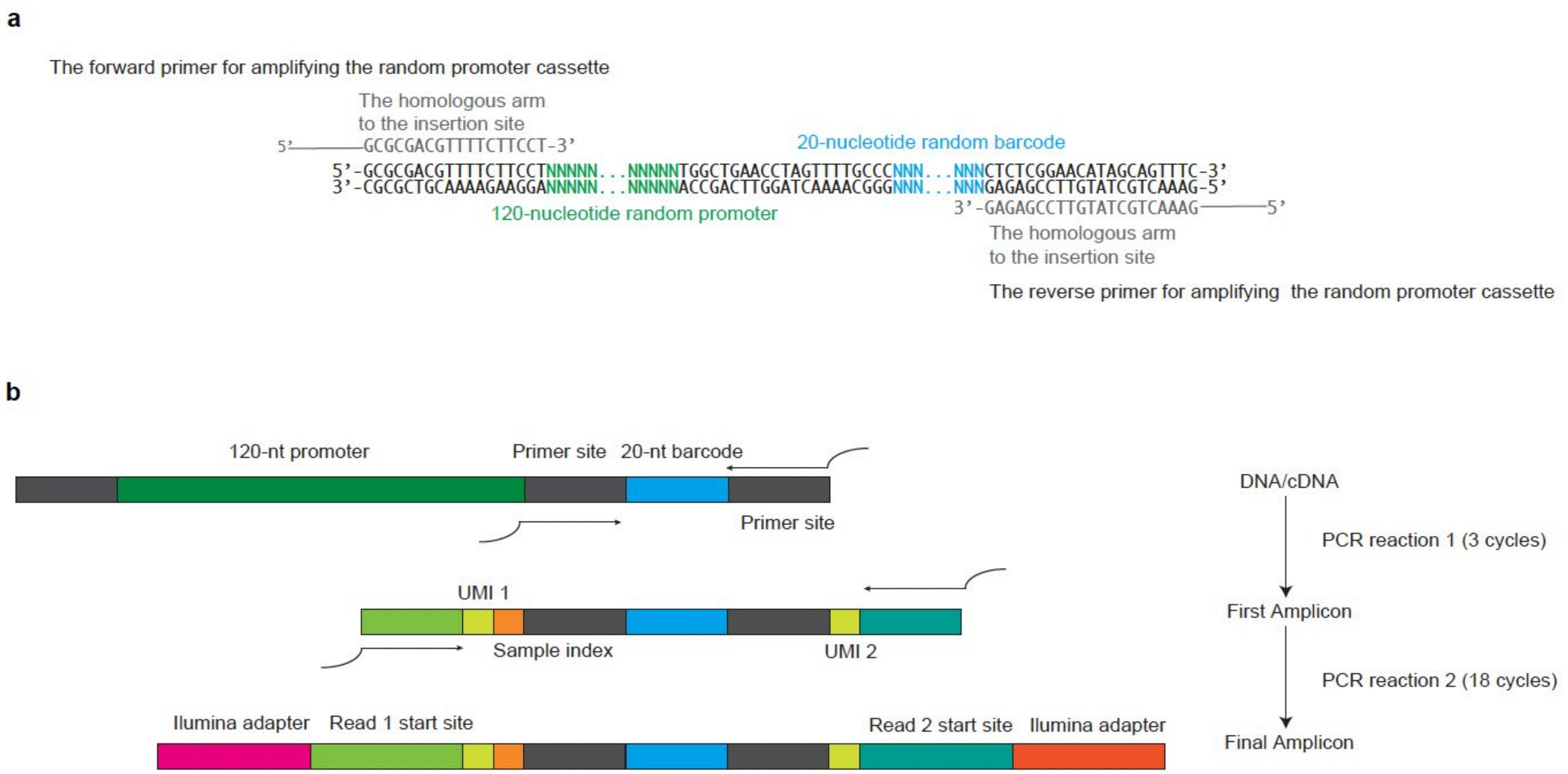

Fig. S1. Construction of the expression cassette and sequencing library. (**a**) The synthesized cassette including the random promoter, random barcode, and invariant primer sequences. (**b**) The two-step PCR procedure for constructing the sequencing library.

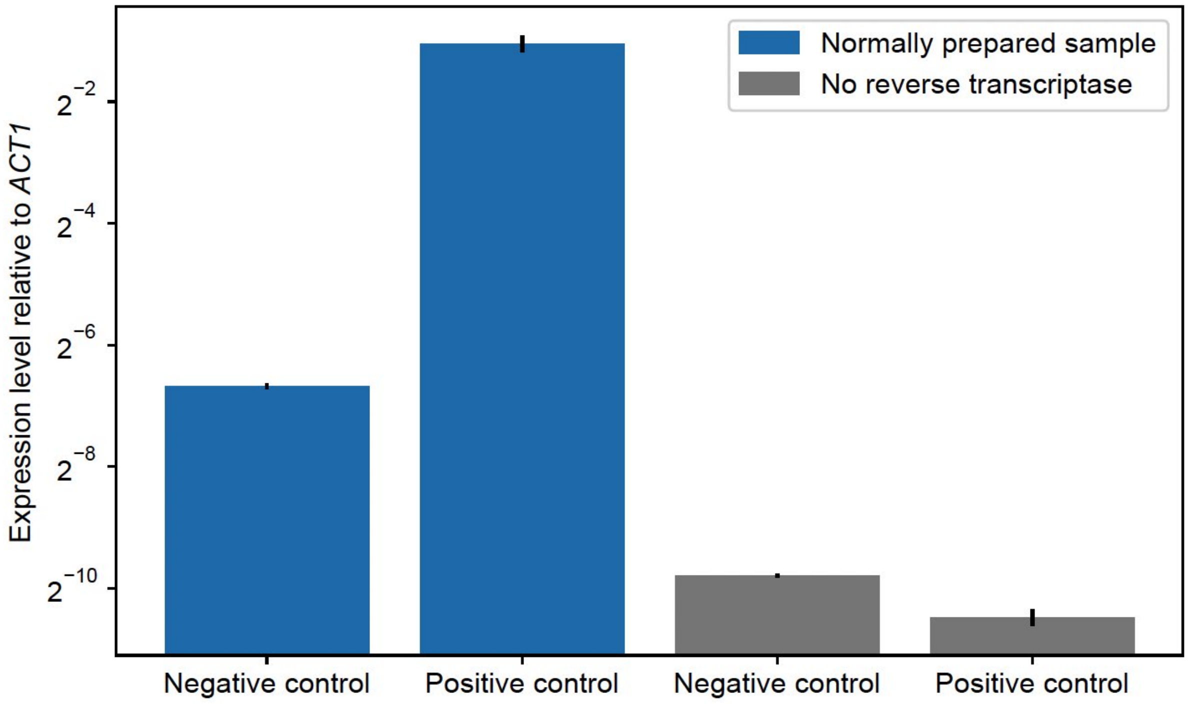

Fig. S2. Expression levels of the positive and negative controls at the chosen integration site. Expression levels are measured by RT-qPCR and shown as ratios to the expression level of the house-keeping gene *ACT1*. The left two columns show the results from normally prepared samples whereas the right two columns show the results from samples without reverse transcriptase. The bar height shows the mean, and the error bar shows the standard error estimated from three technical repeats of the control of interest and three technical repeats of the house-keeping gene. Source data are provided as a Source Data file.

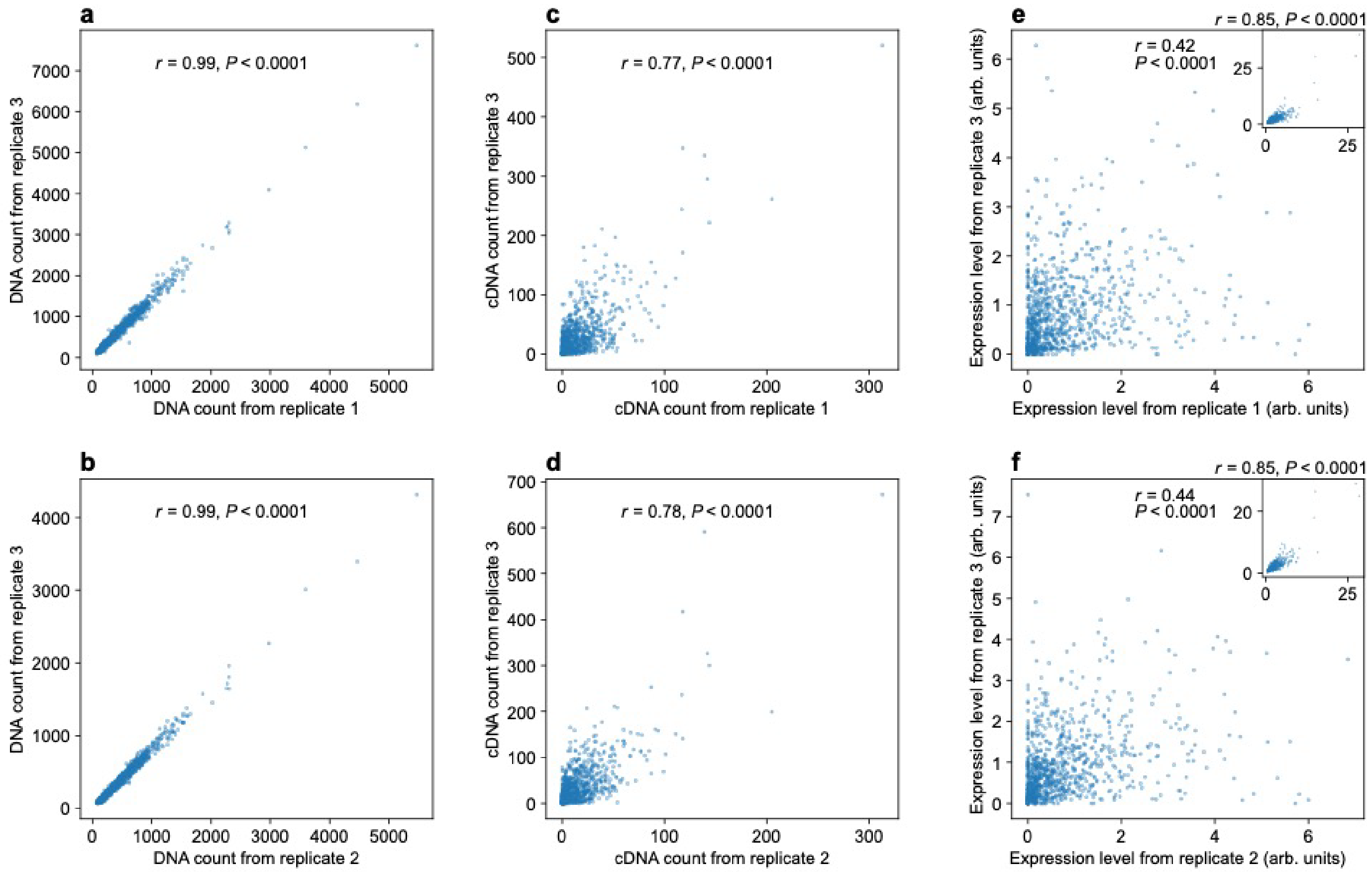

Fig. S3. Between-replicate correlations in barcode DNA count, cDNA count, and expression level in YPD. (**a**) Comparing DNA counts between replicates 1 and 3. (**b**) Comparing DNA counts between replicates 2 and 3. (**c**) Comparing cDNA counts between replicates 1 and 3. (**d**) Comparing cDNA counts between replicates 2 and 3. (**e**) Comparing expression levels between replicates 1 and 3. (**f**) Comparing expression levels between replicates 2 and 3. For clarity, only 1000 randomly sampled barcodes are shown. Pearson's correlation (r) and associated P-value based on all barcodes are presented. The inset in (e) and (f) shows the expression levels of 1% of genotypes with the highest cDNA counts and associated statistics. All P-values are from two-tailed tests.

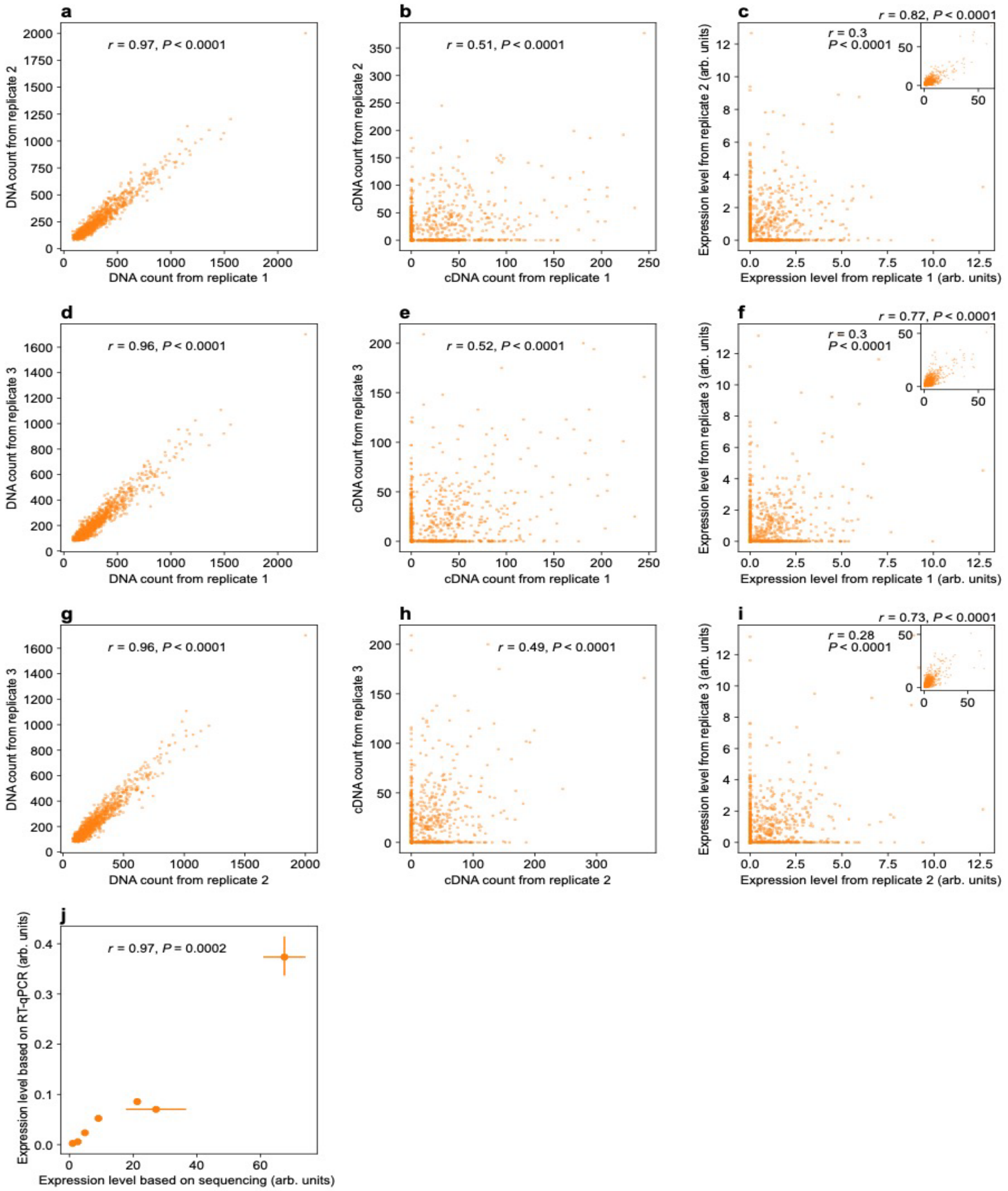

Fig. S4. Between-replicate correlations in barcode DNA count, cDNA count, and expression level in SCD. (**a−c**) Comparing DNA counts (a), cDNA counts (b), and expression levels (c)

between replicates 1 and 2. (**d–f**) Comparing DNA counts (d), cDNA counts (e), and expression levels (f) between replicates 1 and 3. (**g–i**) Comparing DNA counts (g), cDNA counts (h), and expression levels (i) between replicates 2 and 3. For clarity, only 1000 randomly sampled barcodes are shown. Pearson's correlation (*r*) and associated *P*-value based on all barcodes are presented. The inset in (c), (f), and (i) shows the expression levels of 1% of genotypes with the highest cDNA counts and associated statistics. (**j**) Expression levels measured by bulk sequencing are strongly correlated with those measured by RT-qPCR in five reconstructed genotypes, a randomly picked negative control, and a randomly picked positive control. Mean expressions and standard errors are shown by dots and error bars, respectively. Pearson's correlation (*r*) and the associated *P*-value between the two measurements are presented. All *P*-values are from two-tailed tests. Source data are provided as a Source Data file.

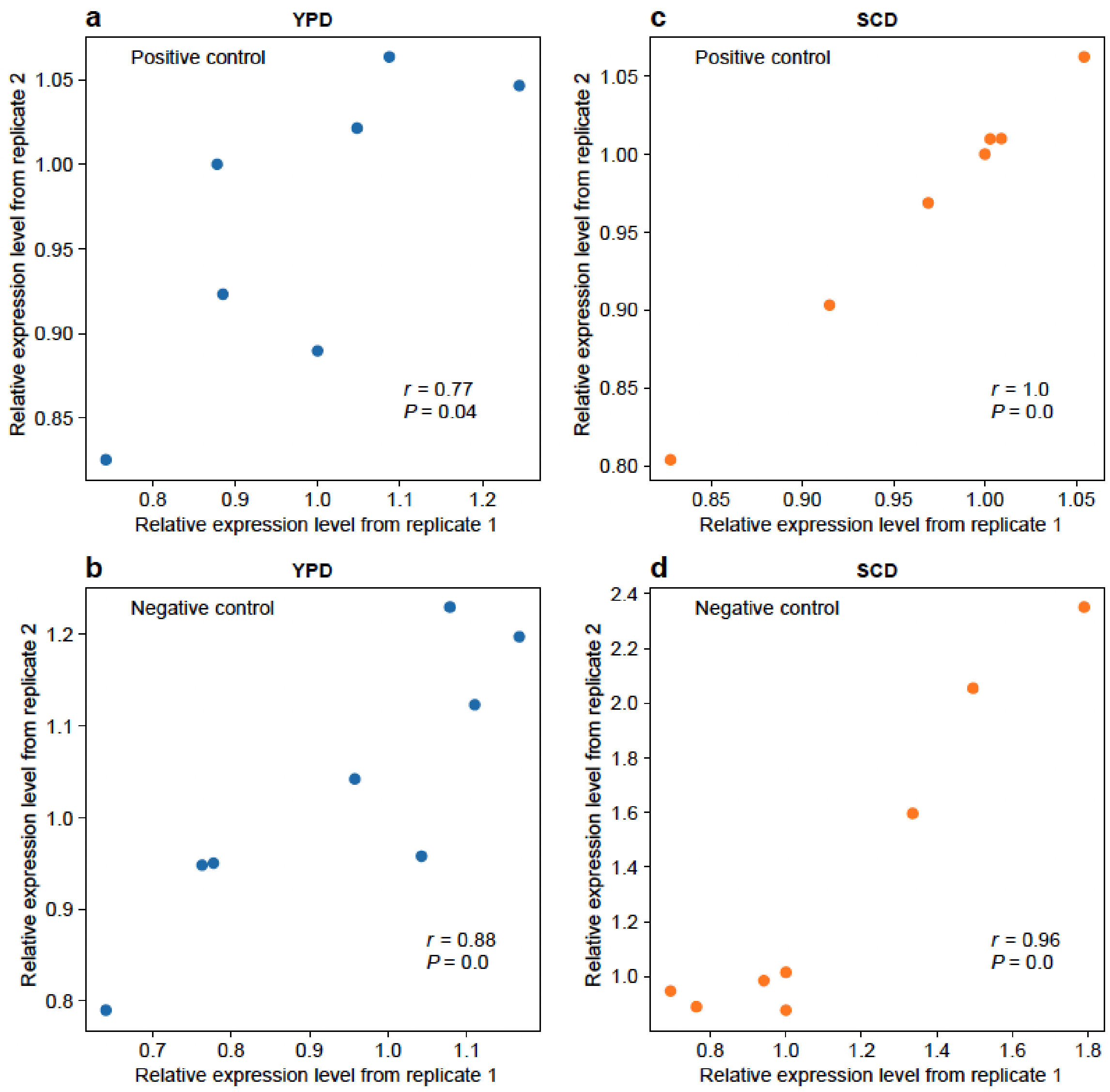

Fig. S5. Between-replicate expression correlation of barcodes of the positive control or negative control. (**a**) Relative expression levels of barcodes of the positive control in YPD. (**b**) Relative expression levels of barcodes of the negative control in YPD. (**c**) Relative expression levels of barcodes of the positive control in SCD. (**d**) Relative expression levels of barcodes of the negative control in SCD. The relative expression level is the expression level of a barcode divided by the median expression level of all barcodes of the positive (or negative) control. Each dot represents a barcode. Pearson's correlation *r* and its associated *P*-value are presented. All *P*-values are from two-tailed tests. Source data are provided as a Source Data file.

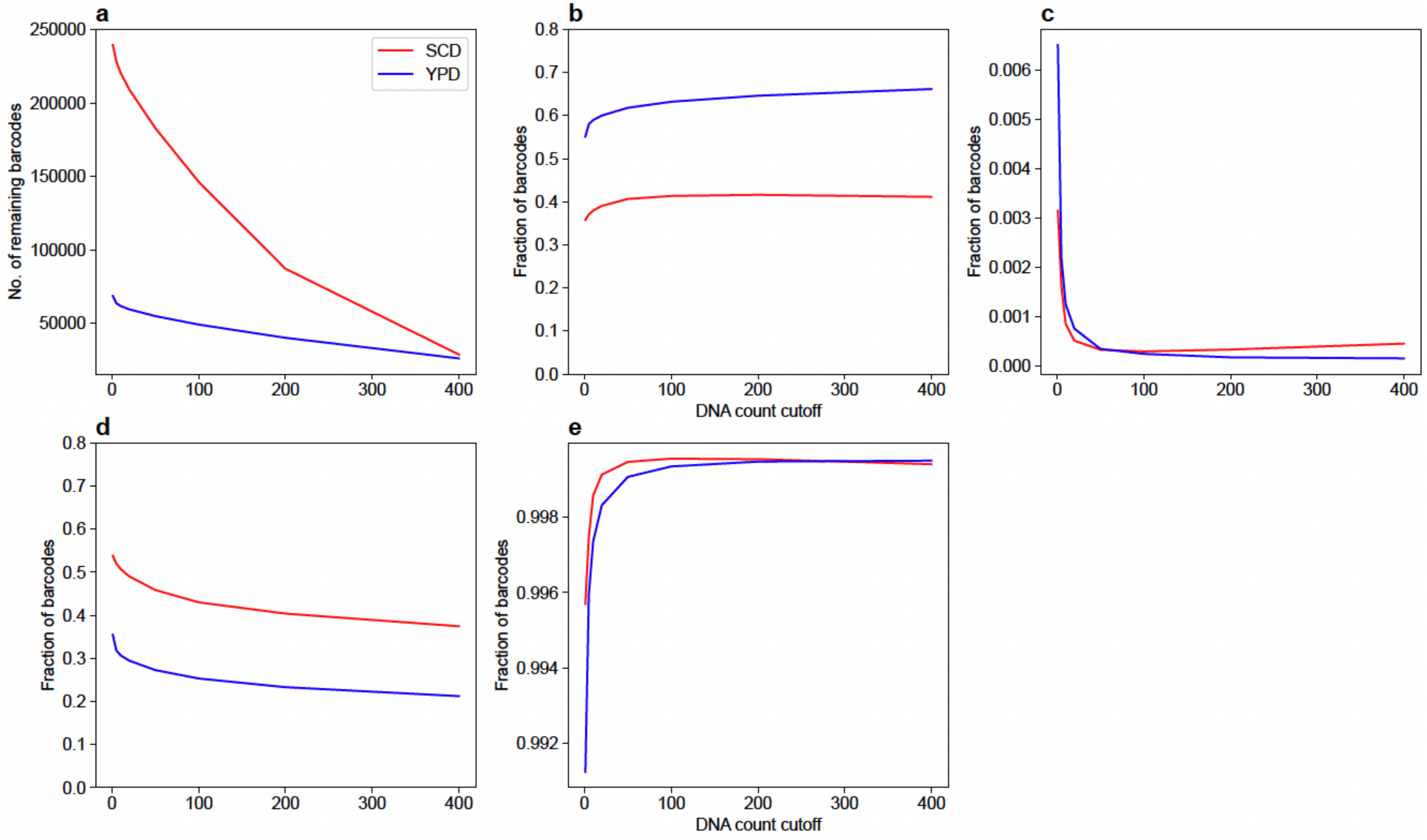

Fig. S6. General robustness of results to higher DNA count cutoffs than the one used in the main analysis. (**a**) Number of remaining random promoters or barcodes. (**b**) Fraction of barcodes with significantly higher expressions than the negative control. (**c**) Fraction of barcodes with significantly higher expressions than the reference (median expression level of yeast native genes). (**d**) Fraction of barcodes with significantly lower expressions than the negative control. (**e**) Fraction of barcodes with significantly lower expressions than the reference. One sample *t*-test is used, followed by multiple-testing corrections at FDR = 0.05. Source data are provided as a Source Data file.

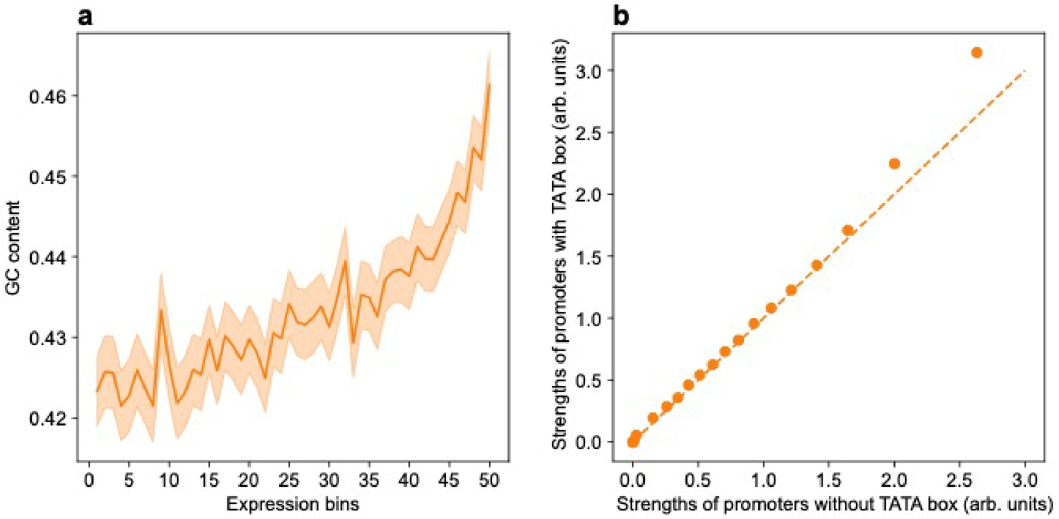

Fig. S7. Sequence features associated with the random promoter strength in SCD. (**a**) The GC content in a random promoter increases with the promoter strength. The promoters are divided into 50 equal-size bins by their strengths. Shown are the mean GC content of each bin, with the shaded area indicating the 95% confidence intervals of the mean GC content. Spearman's rank correlation between the promoter strength and GC content for unbinned data is $\rho = 0.14$ ($P = 5.6\times10^{-150}$). (**b**) Quantile-quantile plot showing the probability distributions of strengths of promoters with and without TATA boxes. The dots show the 0th, 5th, 10th, …, and 95th percentiles of the data in promoter strength. The strengths of promoters with TATA boxes are significantly higher than those without ($P = 0.02$, Wilcoxon rank-sum test). All *P*-values are from two-tailed tests. Source data are provided as a Source Data file.

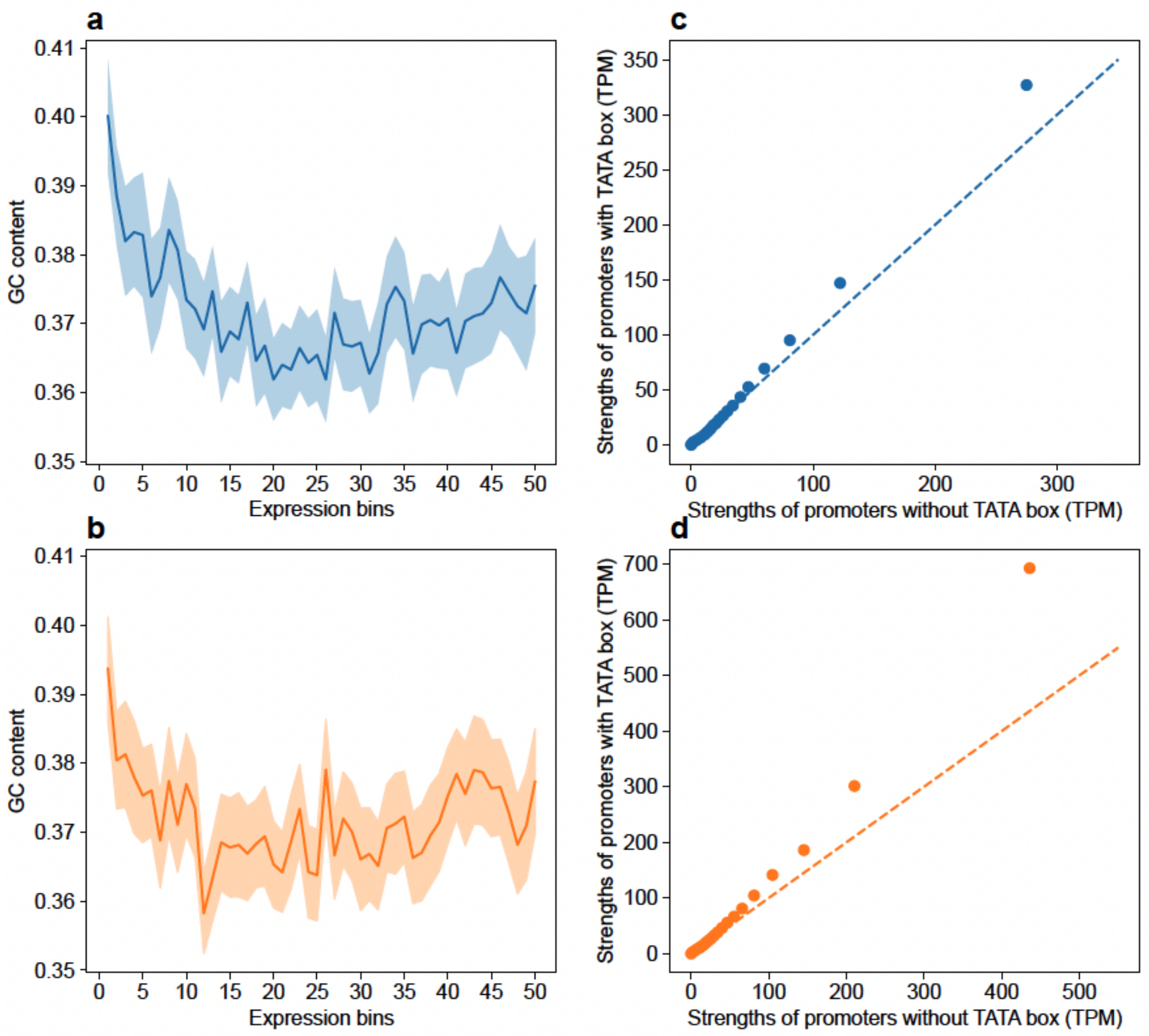

Fig. S8. Sequence features of yeast native promoters. The native promoter is defined as the 500 nucleotides upstream of the start codon. (**a**−**b**) The GC content first decreases and then increases with the native promoter strength in both YPD (a) and SCD (b). Shown are the mean GC content of each bin, with the shaded area indicating the 95% confidence intervals of the mean GC content. Spearman's rank correlation between the promoter strength in YPD and GC content for unbinned data is -0.19 ($P = 2.5\times10^{-25}$) and 0.07 ($P = 1.9\times10^{-5}$) for the weakest 40% and strongest 60% of promoters, respectively. The corresponding correlations in SCD are -0.16 ($P = 1.1\times10^{-13}$) and 0.07 ($P = 7.2\times10^{-6}$) for the weakest 30% and strongest 70% of promoters, respectively. (**c**−**d**) Quantile-quantile plot showing the probability distributions of strengths of promoters in YPD (c) or SCD (d) with and without TATA boxes. The dots show the 0th, 5th, 10th, …, and 95th percentiles of the data in promoter strength. The strengths of promoters with TATA boxes are significantly higher than those without ($P = 0.74$ in YPD and 0.007 in SCD, Wilcoxon rank-sum test). TPM, transcript per million. All *P*-values are from two-tailed tests. Source data are provided as a Source Data file.

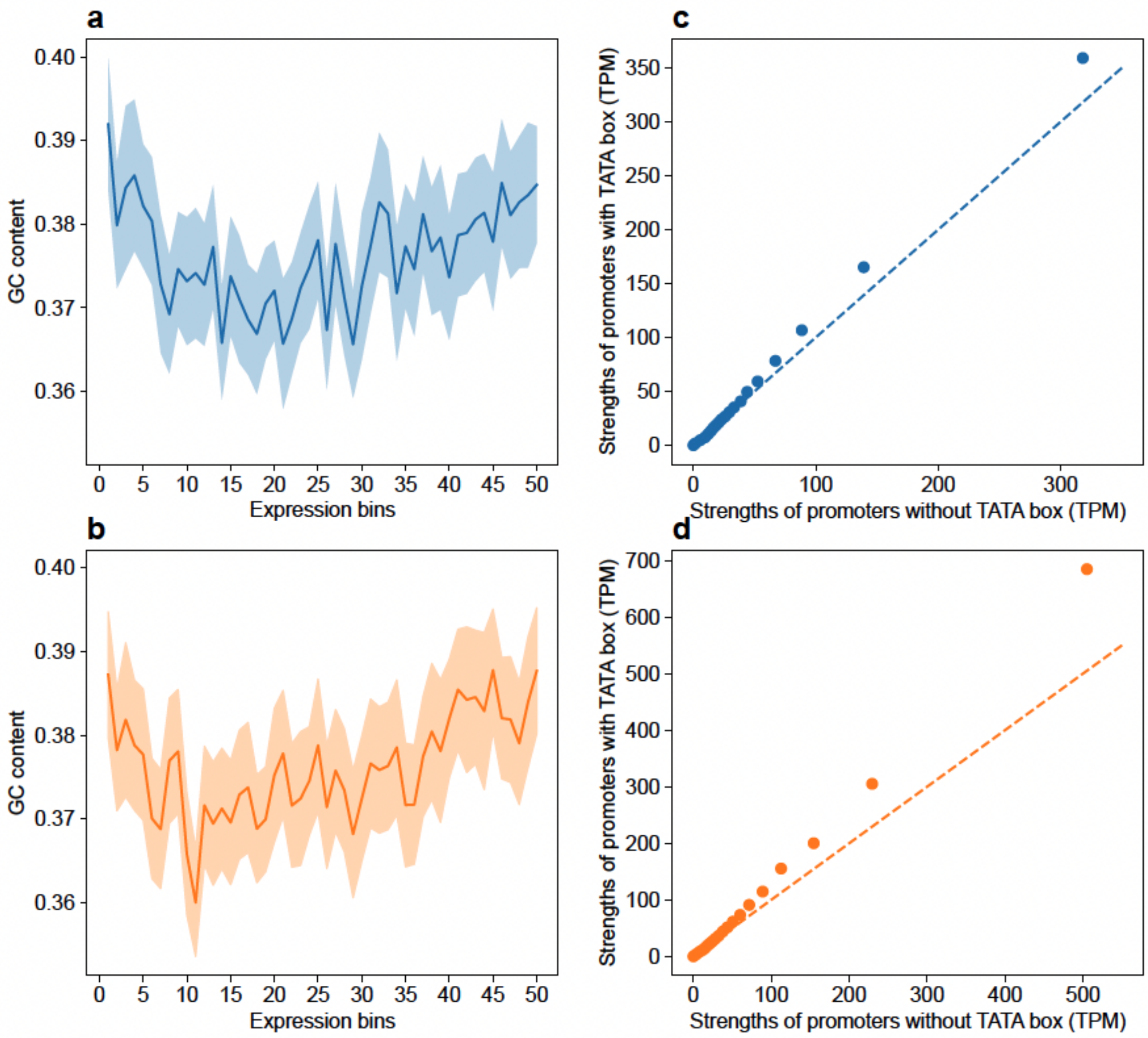

Fig. S9. Sequence features of native promoters. Same as Fig. S8 except that the promoter is defined as 500 nucleotides upstream of the transcription start site. (**a−b**) The GC content first decreases and then increases with the native promoter strength in both YPD (a) and SCD (b). Shown are the mean GC content of each bin, with the shaded area indicating the 95% confidence intervals of the mean GC content. Spearman's rank correlation between the promoter strength in YPD and GC content for unbinned data is -0.13 ($P = 5.8\times10^{-11}$) and 0.08 ($P = 1.0\times10^{-6}$) for the weakest 40% and strongest 60% of promoters, respectively. The corresponding correlations in SCD are -0.11 ($P = 1.0\times10^{-6}$) and 0.10 ($P = 5.3\times10^{-10}$) for the weakest 30% and strongest 70% of promoters, respectively. (**c−d**) Quantile-quantile plot showing the probability distributions of strengths of promoters in YPD (c) or SCD (d) with and without TATA boxes. The dots show the 0th, 5th, 10th, …, and 95th percentiles of the data in promoter strength. The strengths of promoters with TATA boxes are significantly higher than those without ($P = 0.50$ in YPD and 0.002 in SCD, Wilcoxon rank-sum test). TPM, transcript per million. All P-values are from two-tailed tests. Source data are provided as a Source Data file.

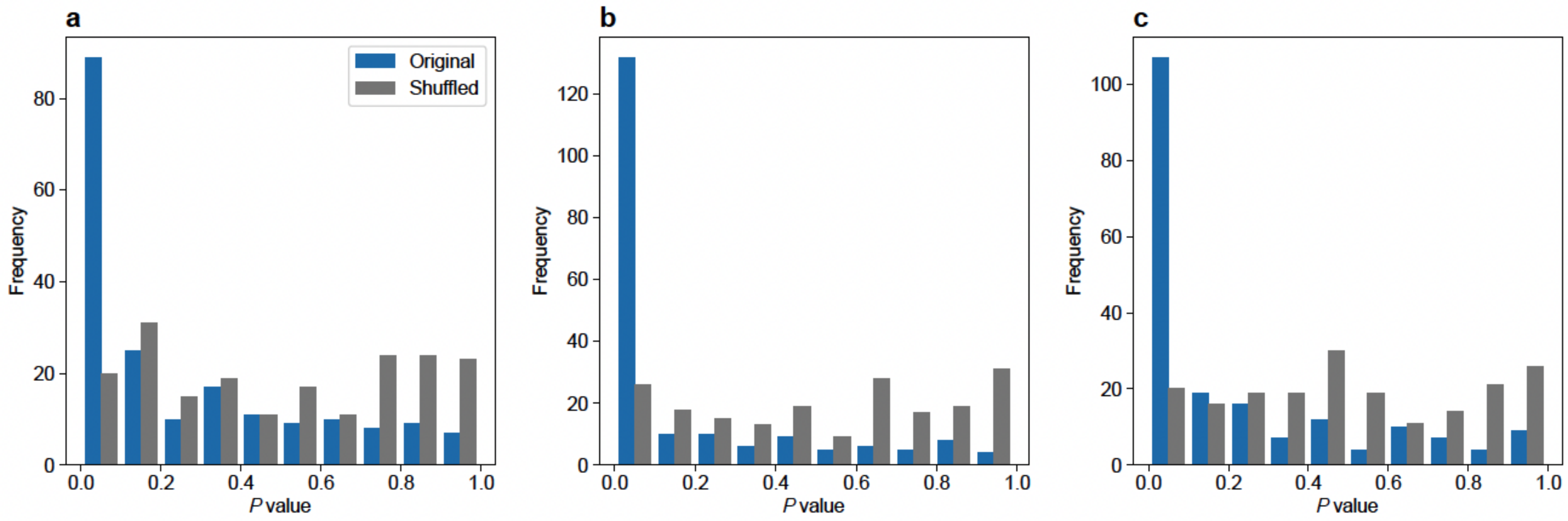

Fig. S10. Frequency distribution of two-tailed *P*-values from Wilcoxon rank-sum tests of equal activities between random promoters with binding sites of a particular TF (on the forward strand) and those without. The blue color indicates *P*-values from the original data, whereas the grey color indicates *P*-values from the data in which the promoter activity is randomly shuffled among promoters. (**a**) Results from SCD when TFBSs are on the forward strand. (**b**) Results from YPD when TFBSs are on the reverse strand. (**c**) Results from SCD when TFBSs are on the reverse strand. All *P*-values are from two-tailed tests. Source data are provided as a Source Data file.

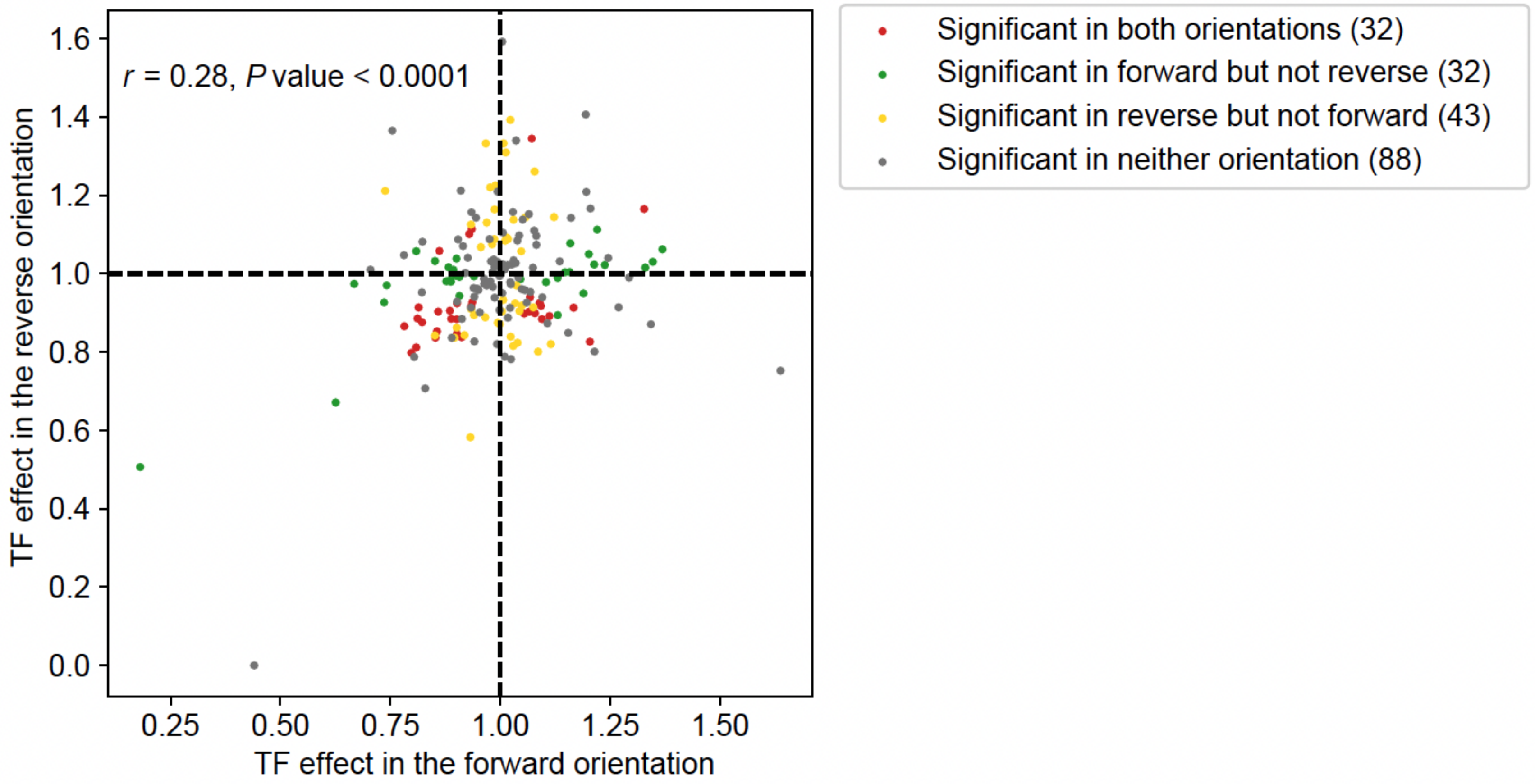

Fig. S11. TF effects on the forward and reverse orientations in SCD. The effect of a TF in an orientation is measured by the median activity of promoters with the binding sites of the TF in the orientation concerned relative to that of promoters without the binding sites in the orientation concerned. Each dot represents a TF, and colors indicate results from two-tailed Wilcoxon rank-sum tests. Number of TFs belonging to each of the four categories is shown in the parentheses. The *P*-value shown is from a two-tailed test. Source data are provided as a Source Data file.

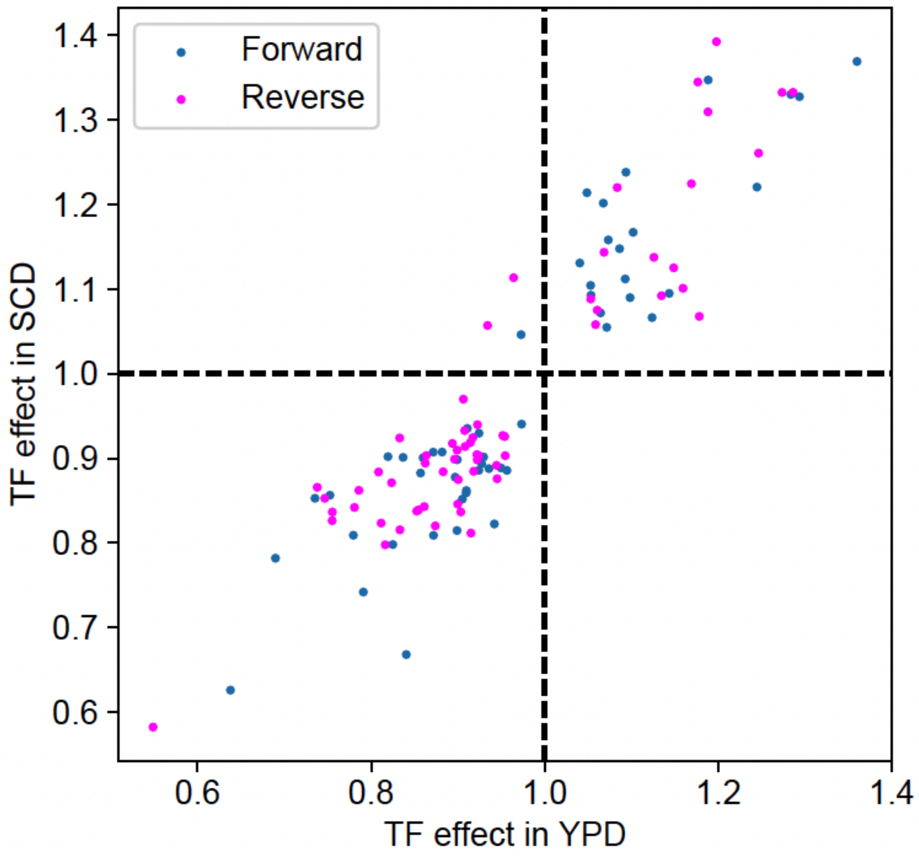

Fig. S12. Environment-specific effects of TFs. The effect of a TF in an orientation is measured by the median activity of promoters with the binding sites of the TF in the orientation concerned relative to that of promoters without the binding sites in the orientation concerned. Each dot represents a TF, and the color indicates the orientation considered. All TFs considered here have significant effects on promoter activity in both YPD and SCD. Source data are provided as a Source Data file.

Strongest random promoter in YPD

Yhp1 binding site

5’-AAAGGCTGTTTCATCAGCCGTCCGTATAATGGTAGCAAGTGAAGGACTTTTTGGCATAATTGAGCGTAGGACCTATAGCGTACAGGTTATTGGACGCATGCCAGAATTTGTGTATGATGA-3’
3’-TTTCCGACAAAGTAGTCGGCAGGCATATTACCATCGTTCACTTCCTGAAAAACCGTATTAACTCGCATCCTGGATATCGCATGTCCAATAACCTGCGTACGGTCTTAAACACATACTACT-5’

Ste12 binding site

Hap2 binding site

Strongest random promoter in SCD

Mot3 binding site

5’-AAGAAGGCAATAGTGTTTGTCGATGCGGTAAATCAAATGCGGGCACAGTCATTACTCGGGATTCATGGCGTAGTGGTTGATGTTTAACGGTAGTATGTTCGTGAAATCTCGCACCAAGCC-3’
3’-TTCTTCCGTTATCACAAACAGCTACGCCATTTAGTTTACGCCCGTGTCAGTAATGAGCCCTAAGTACCGCATCACCAACTACAAATTGCCATCATACAAGCACTTTAGAGCGTGGTTCGG-5’

Rds2 binding site

A strong random promoter in both conditions

Cst6 binding site

5’-TTATTTTAGACGAGATATATGGGAGTACTTTCGACTGTTTAATGCATCTTGGCATCGTGAATCAACAGGGTTTACCTAATCAAAAATTACCGAGAATATTGACGTGTTGGTTATGATGAT-3’
3’-AATAAAATCTGCTCTATATACCCTCATGAAAGCTGACAAATTACGTAGAACCGTAGCACTTAGTTGTCCCAAATGGATTAGTTTTTAATGGCTCTTATAACTGCACAACCAATACTACTA-5’

Phd1 binding site (SMTGCAKN)

Mot3 binding site

Rim101 binding site (TGCCAAG)

Fig. S13. Three strong promoters with sites perfectly matching transcription factor binding sites indicated. For the random promoter that is strong in both conditions, the shared nucleotide for Phd1 binding site and Rim101 binding site is indicated in red color**.**

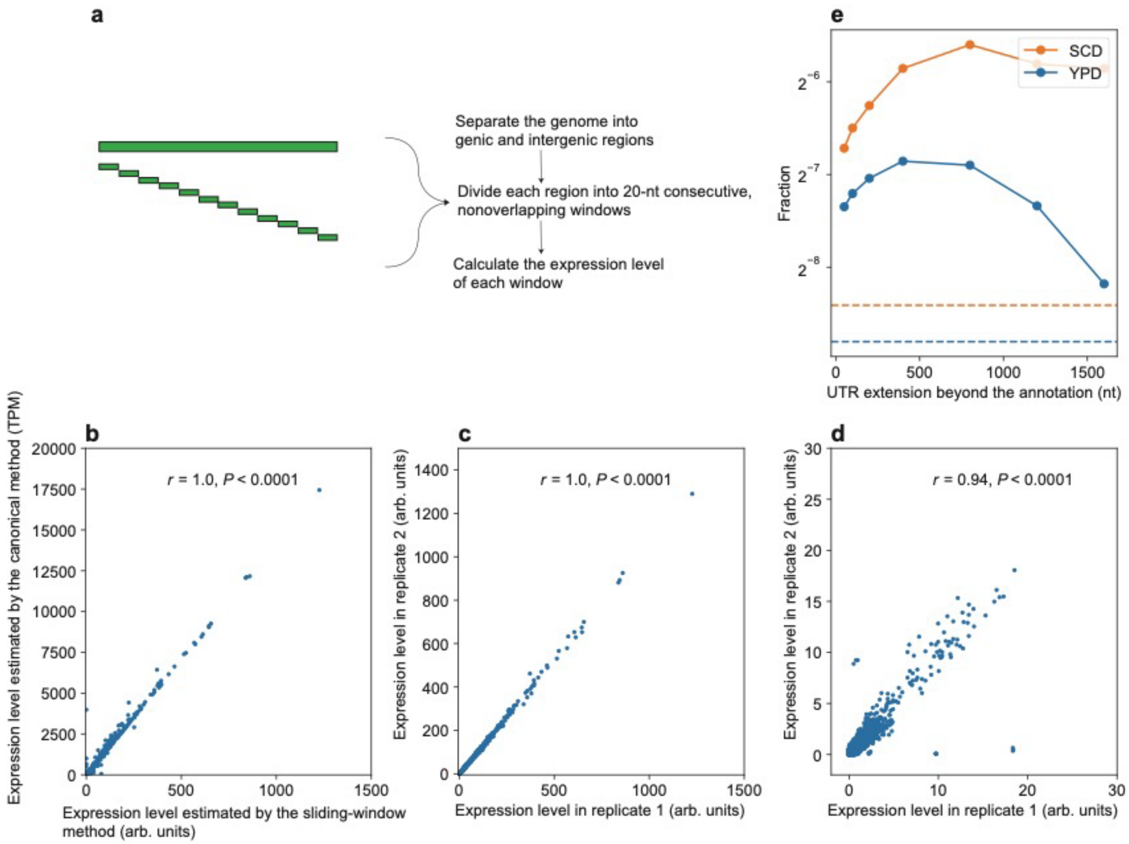

Fig. S14. Intergenic expressions in yeast. (**a**) Diagram explaining the sliding-window method for measuring the expression level of a genic or intergenic region. (**b**) Correlation between expression levels (in YPD) estimated by the canonical method and the sliding-window method for genic regions. Each dot represents a gene. TPM, transcript per million. (**c**) Between-replicate correlation of genic expressions (in YPD) estimated by the sliding-window method. Each dot represents a gene. (**d**) Between-replicate correlation of intergenic expressions (in YPD) estimated by the sliding-window method. Each dot represents an intergenic window. Pearson's correlation (r) and the associated two-tailed P-value are presented. (**e**) Fraction of intergenic windows with significantly higher expressions than the median expression level of yeast genes under different UTR extensions. The blue and orange horizontal lines indicate the fraction of barcodes with significantly higher expressions than the median yeast gene expression in YPD and SCD, respectively. Source data are provided as a Source Data file.

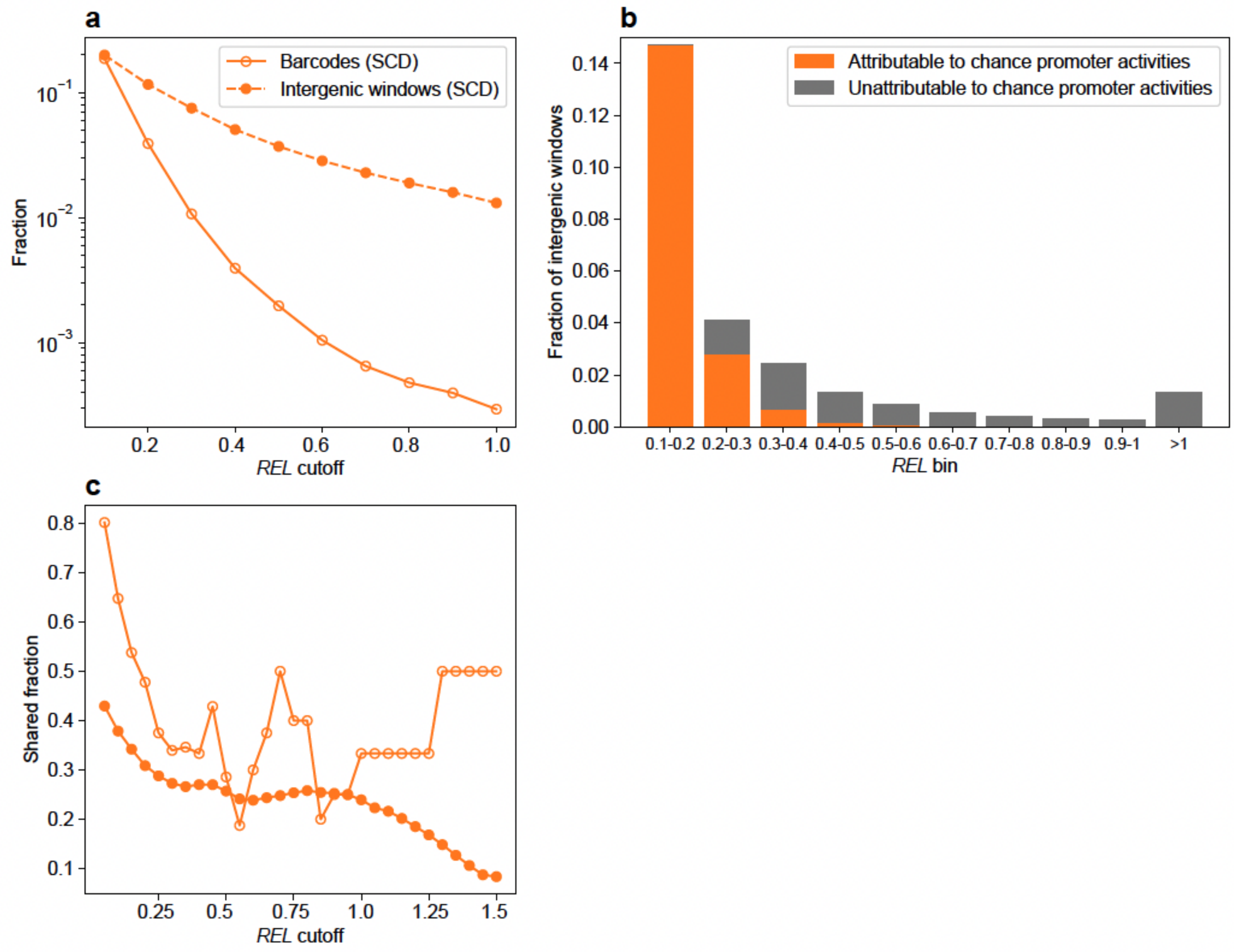

Fig. S15. Intergenic expressions in SCD attributable to chance promoter activities. (**a**) Fractions of barcodes or intergenic windows with *REL*s in SCD significantly higher than various cutoffs. For instance, the bin of 0.6 includes all barcodes or intergenic windows with *REL*s significantly exceeding 0.6. (**b**) Fractions of intergenic windows whose SCD expressions are attributable (orange) or unattributable (grey) to chance promoter activities. For example, the bin of 0.2–0.3 includes intergenic windows with *REL*s significantly higher than 0.2 but not significantly higher than 0.3. Only bins with grey areas are shown. (**c**) Fraction of barcodes or intergenic windows with *REL*s significantly exceeding a cutoff in SCD that have *REL*s significantly exceeding the same cutoff in YPD. Symbols follow (a). Source data are provided as a Source Data file.

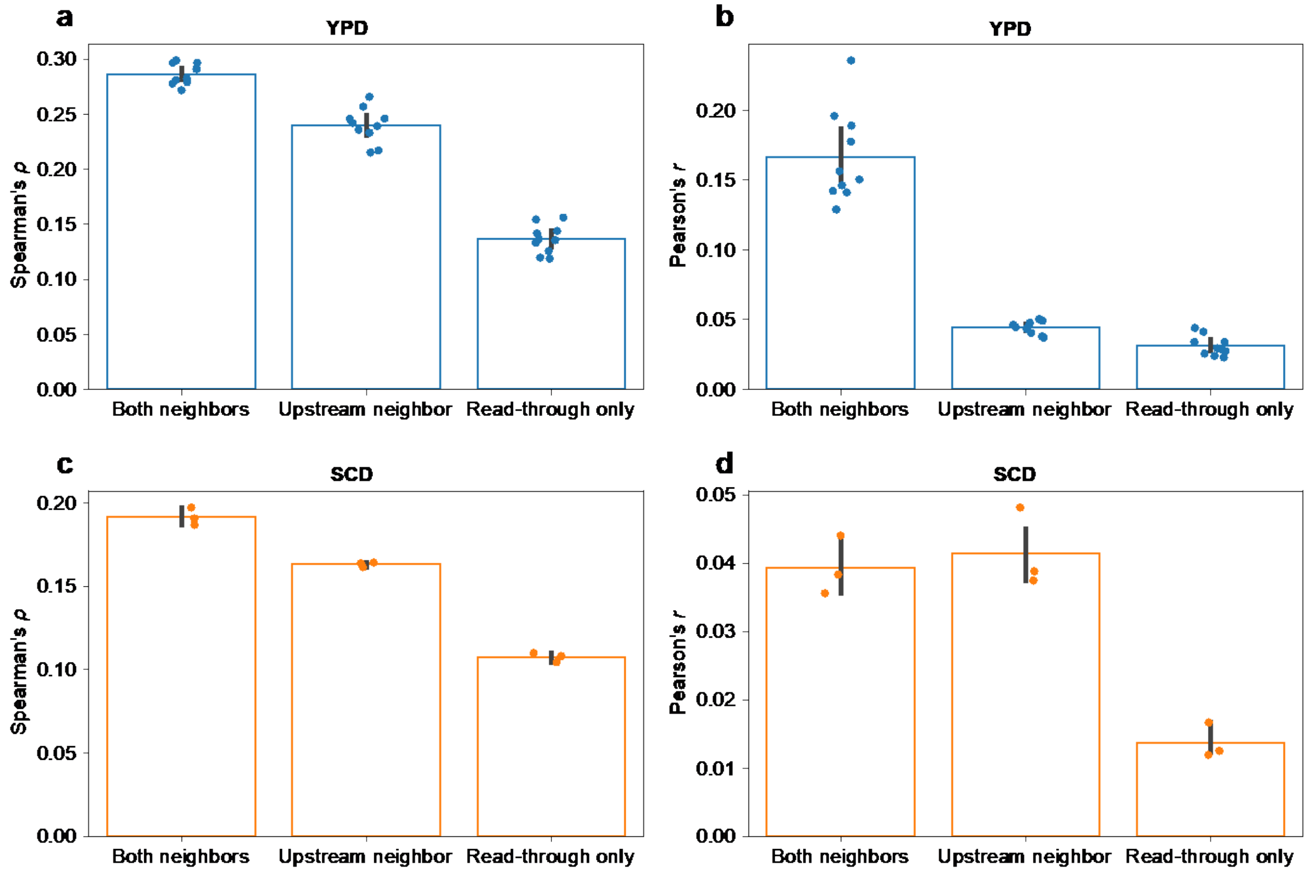

Fig. S16. Correlation between the expression level of an intergenic region and its neighboring gene expression. (**a**) Spearman's rank correlation coefficient in YPD. (**b**) Pearson's linear correlation coefficient in YPD. (**c**) Spearman's rank correlation coefficient in SCD. (**d**) Pearson's linear correlation coefficient in SCD. The three bars in each panel respectively correspond to the three ways of examining neighboring gene expressions outlined in the main text. Bars show the means, and error bars show 95% confidence intervals. Each dot represents the result from one RNA-seq replicate. Source data are provided as a Source Data file.

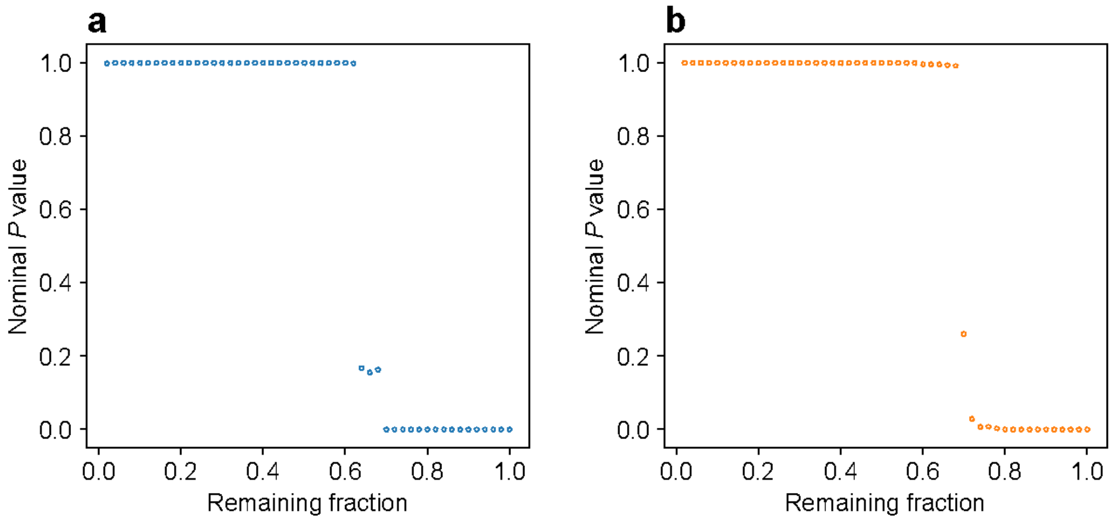

Fig. S17. Nominal (one-tailed) *P* values from tests of the equality between the remaining intergenic regions (after removing those with the highest neighboring gene expressions) and the same number of randomly picked intergenic regions in neighboring gene expression levels in YPD (a) or SCD (b). Each test was performed by 1000 sets of randomly picked intergenic regions. Source data are provided as a Source Data file.

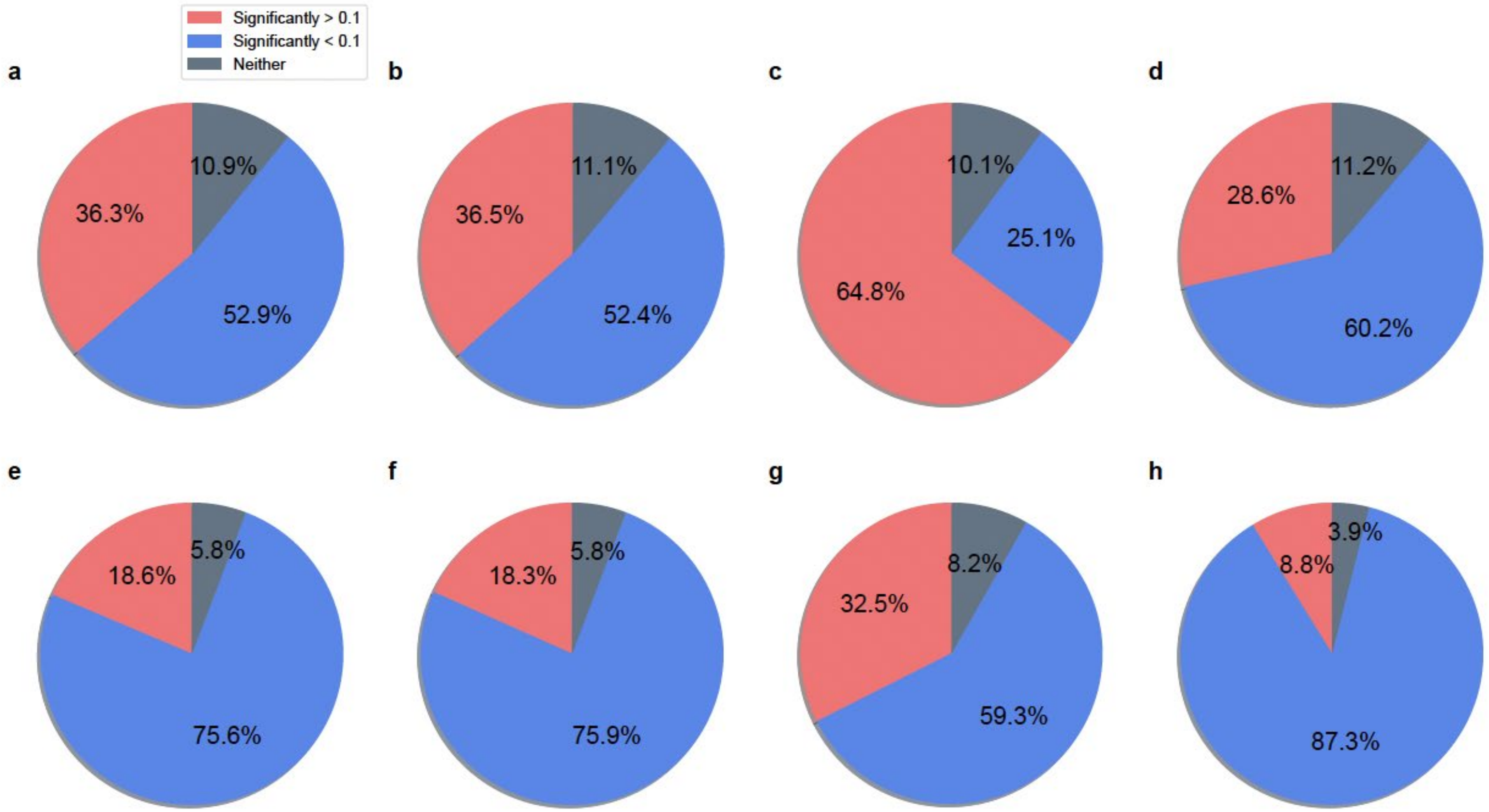

Fig. S18. Environment-specific expressions of barcodes. (**a−d**) Profiling barcodes by comparing their relative expression levels (*REL*s) in YPD with 0.1, for all barcodes in YPD (a), barcodes shared between YPD and SCD (b), barcodes having significantly higher *REL*s than 0.1 in SCD (c), and barcodes with significantly lower *REL*s than 0.1 in SCD (d). (**e−h**) Profiling barcodes by comparing their relative expression levels (*REL*s) in SCD with 0.1, for all barcodes in SCD (e), barcodes shared between YPD and SCD (f), barcodes having significantly higher *REL*s than 0.1 in YPD (g), and barcodes with significantly lower *REL*s than 0.1 in YPD (h). Source data are provided as a Source Data file.

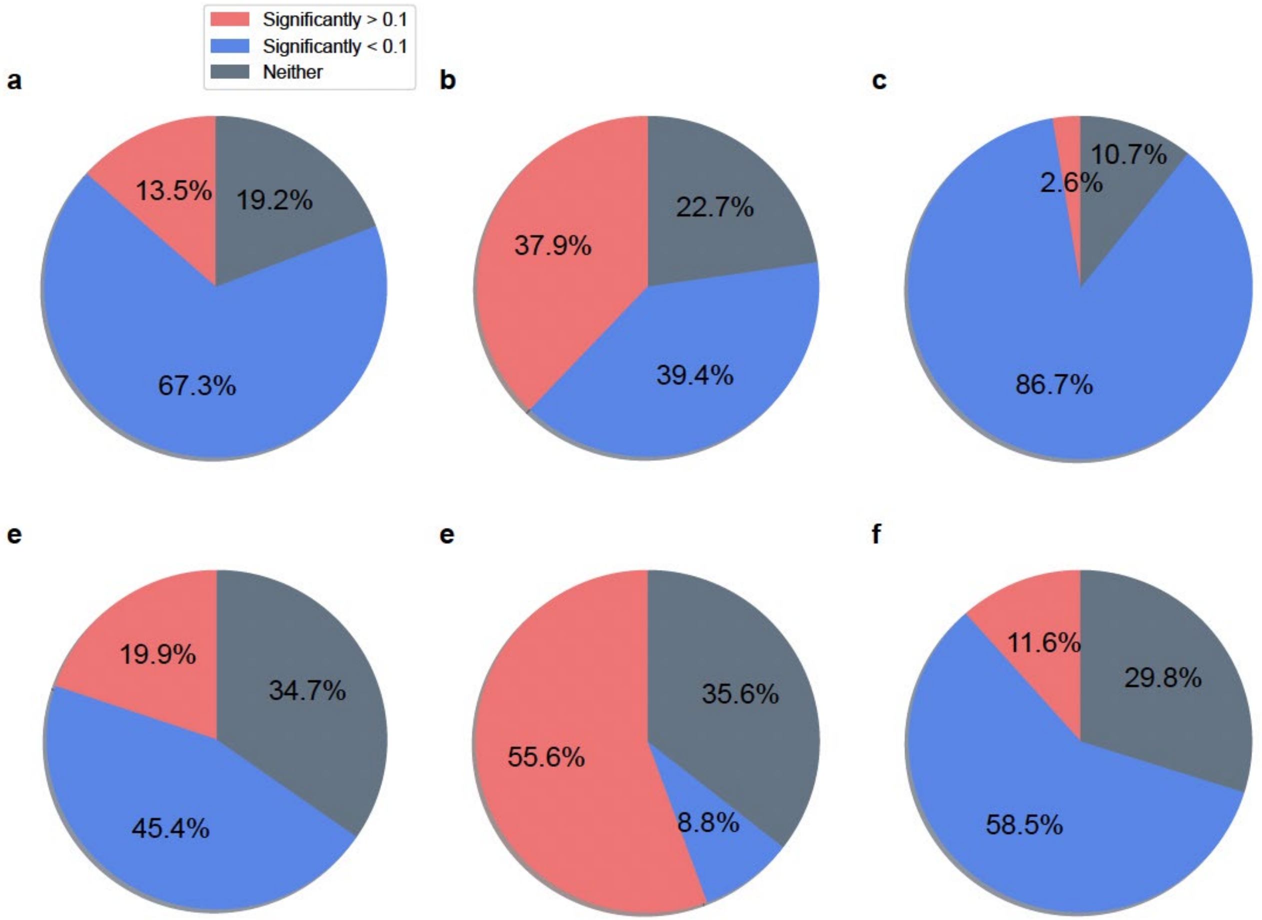

Fig. S19. Environment-specific expressions of intergenic regions. (**a−c**) Profiling intergenic regions by comparing their relative expression levels (*REL*s) in YPD with 0.1, for all intergenic regions in YPD (a), intergenic regions having significantly higher *REL*s than 0.1 in SCD (b), and intergenic regions with significantly lower *REL*s than 0.1 in SCD (c). (**d−f**) Profiling intergenic regions by comparing their *REL*s in SCD with 0.1, for all intergenic regions in SCD (d), intergenic regions having significantly higher *REL*s than 0.1 in YPD (e), and intergenic regions with significantly lower *REL*s than 0.1 in YPD (f). Source data are provided as a Source Data file.

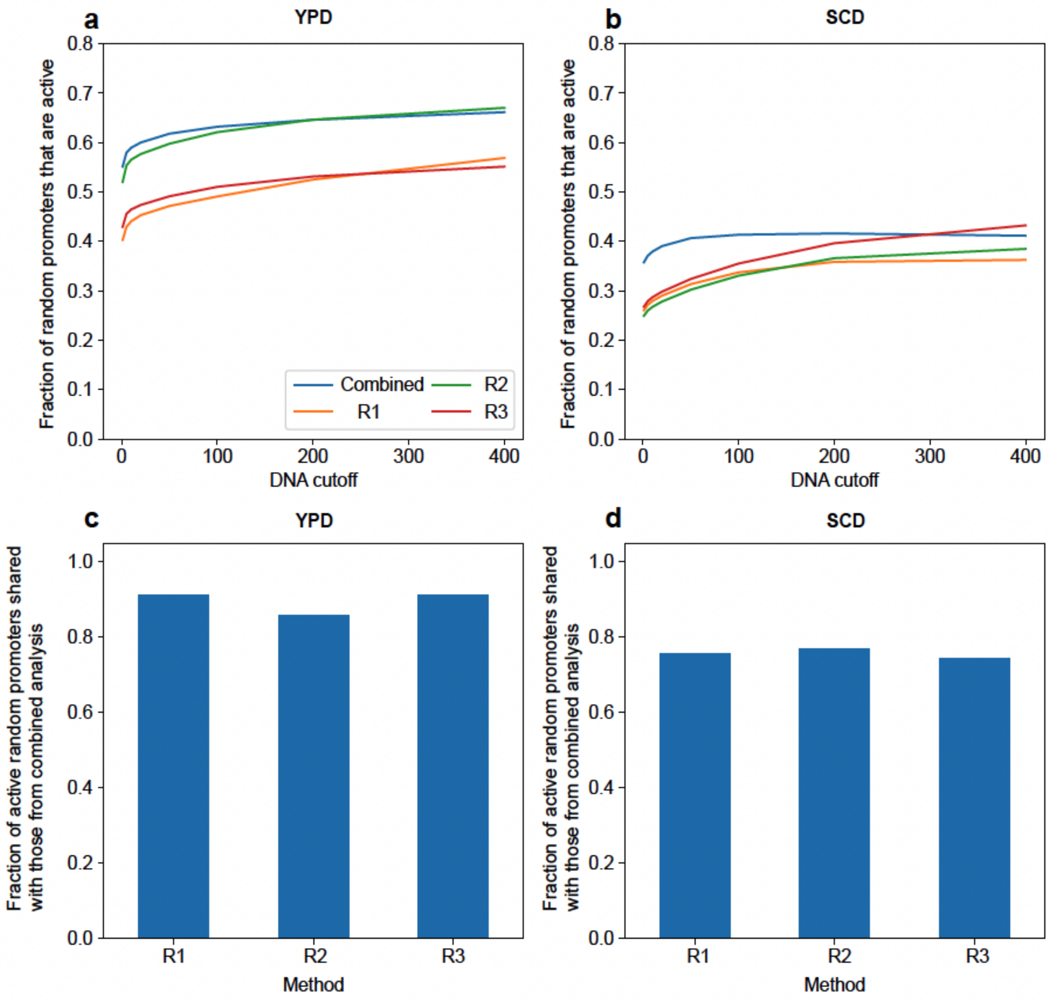

Fig. S20. Comparison of different estimates of the fraction of active random promoters, which have activities significantly greater than the negative control. "Combined" refers to the combined analysis of the data from three biological replicates, whereas R1 to R3 refer to the individual analysis of the data from each replicate. (**a-b**) Fraction of random promoters that are active in YPD (a) and SCD (b), respectively. One sample *t*-test is used, followed by multiple-testing corrections at FDR = 0.05. (**c-d**) Fraction of discovered active random promoters in a replicate that overlap with those discovered by the combined analysis. Source data are provided as a Source Data file.

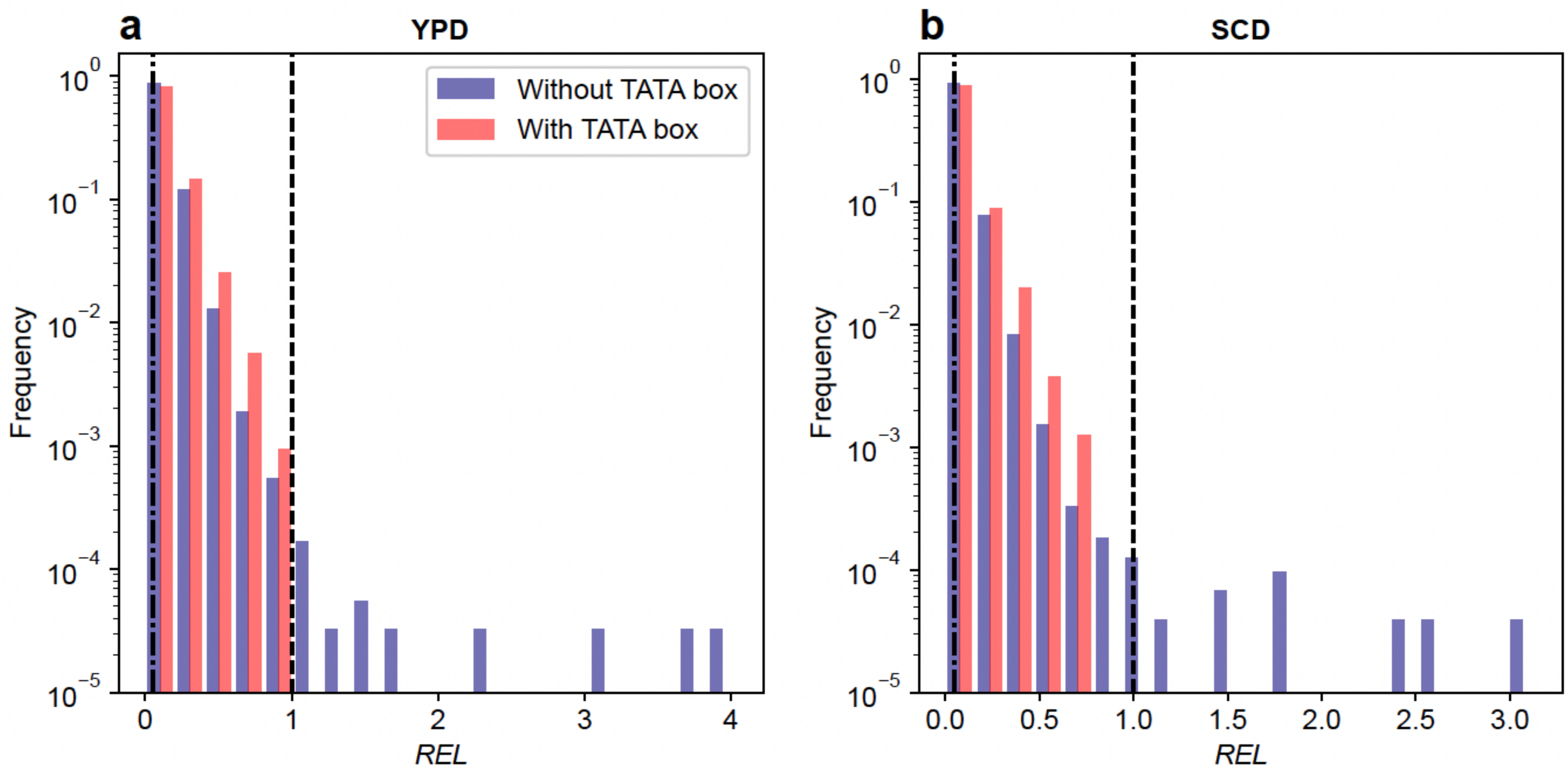

Fig. S21. Expression distribution of barcodes associated with random promoters with TATA boxes in YPD (a) or SCD (b). Barcode expression level is shown relative to the reference, which is the median expression level of yeast native genes. The vertical dash-dot line indicates the mean of the negative control (no promoter) and the vertical dashed line indicates the mean of the reference. Source data are provided as a Source Data file.
